# Enzymatic production of a solvent-free menthyl butyrate via response surface methodology catalyzed by a novel thermostable lipase from *Geobacillus zalihae*

**DOI:** 10.1080/13102818.2014.978220

**Published:** 2014-11-14

**Authors:** Roswanira Abdul Wahab, Mahiran Basri, Raja Noor Zaliha Raja Abdul Rahman, Abu Bakar Salleh, Mohd Basyaruddin Abdul Rahman, Naz Chaibakhsh, Thean Chor Leow

**Affiliations:** ^a^ Department of Chemistry, Faculty of Science, Universiti Teknologi Malaysia, 81310Skudai, Johor, Malaysia; ^b^ Department of Chemistry, Faculty of Science, Universiti Putra Malaysia, 43400Serdang, Selangor, Malaysia; ^c^ Institute of Bioscience, Universiti Putra Malaysia, 43400UPM Serdang, Selangor, Malaysia; ^d^ Department of Cell and Molecular Biology, Faculty of Biotechnology and Biomolecular Sciences, Universiti Putra Malaysia, 43400Serdang, Selangor, Malaysia; ^e^ Department of Chemistry, Faculty of Science, University of Guilan, Rasht, Iran; ^f^ Enzyme and Microbial Technology Research Centre, Universiti Putra Malaysia, 43400 Serdang, Selangor, Malaysia

**Keywords:** T1 lipase, esterification, *Geobacillus zalihae*, central composite rotatable design

## Abstract

Most substrate for esterification has the inherent problem of low miscibility which requires addition of solvents into the reaction media. In this contribution, we would like to present an alternative and feasible option for an efficient solvent-free synthesis of menthyl butyrate using a novel thermostable crude T1 lipase. We investigated the effects of incubation time, temperature, enzyme loading and substrate molar ratio and determined the optimum conditions. The high conversion of menthyl butyrate catalyzed by crude T1 lipase in a solvent-free system is greatly affected by temperature and time of the reaction media. The highest yield of menthyl butyrate was 99.3% under optimized conditions of 60 °C, incubation time of 13.15 h, 2.53 mg, 0.43% (w/w) enzyme to substrate ratio and at molar ratio of butyric anhydride/menthol 2.7:1. Hence, the investigation revealed that the thermostable crude T1 lipase successfully catalyzed the high-yield production of menthyl butyrate in a solvent-free system. The finding suggests that the crude T1 lipase was a promising alternative to overcome shortcomings associated with solvent-assisted enzymatic reactions.

## Introduction

It has been reported that most substrate for esterification carries the inherent problem of low miscibility which requires the addition of organic solvents into the reaction media. Nevertheless, there are undesirable shortcomings that are associated with the use of solvents for instance, the costs of separation of solvents and products, as well as the presence of harmful residual substances in the final product that could be harmful to human health.[1] In recent years, the use of hydrolytic enzymes such as proteases, esterases and peptidases in organic synthesis has been used in the scientific and industrial environments to address the above problems. The versatile enzyme-catalyzed esterification reactions can be carried out in many types of media [2], as these biocatalysts have the added advantages of having high activity in both water and organic solvents, and the ability to convert a large number of substrates with high stereospecificity.[3] Several researchers reported enzymatic synthesis of esters in organic solvent-free systems in an attempt to make the processes feasible.[4–7] Catalysis in a solvent-free system offers the benefits of minimizing environmental impact by avoiding the use of flammable organic and toxic solvents. The manufacturing cost could be considerably reduced by the simpler technique and lack of down-stream processing, as well as fewer steps of product purification.[8] Also, when substrate alone promotes sufficient homogeneity for the reaction system, the use of solvent-free systems is the more interesting medium to maintain the effectiveness of an interface acting biocatalyst such as lipases, specifically in terms of interfacial factor.[9] Another major requirement for the use of enzyme for industrial processes is thermal stability, as thermal denaturation is a common cause of enzyme deactivation.[10] This problem can be solved by the use of thermostable enzymes as these enzymes allow reactions to progress at higher temperatures, thereby, accelerating conversion rates, substrate solubility and improve miscibility in media.

The experimental conditions in organic synthesis are also of a major concern as all enzymatic reactions are influenced by those conditions. Due to the nonlinear manner, uncertainties and complicated structure of biotechnological practices, predictions of the effects of independent variables on the product and rate of reaction become very difficult. Moreover, the task of finding the optimal conditions to increase efficiency of bioprocesses is almost impossible due to the lack of appropriate deterministic mathematical variables of the product and rate of the reaction. Sensitivity of enzyme structure to variables such as temperature, reaction time, substrate molar ratio and activator or inhibitor concentrations could potentially increase the complexity of the models.[8] To address this matter, response surface methodology (RSM) is a statistical tool of choice for researchers to optimize [11] multiple variables and predict better performance conditions.[12] The method allows determination of optimum conditions under predetermined reaction preferences, such as high product yield at the lowest cost and/or with the least number of experiments. RSM uses quantitative data in experimental design to conclude and simultaneously solve multivariate equations in order to optimize processes or products.[13] RSM has been successfully applied to study and optimize enzymatic syntheses of various esters, [14,15] as this technique is more rapid and less expensive than the conventional one-variable-at-a-time or full factorial experiment when gathering research information.[16,17]

Previously, we described that the thermostable T1 lipase, isolated from *Geobacillus zalihae*, was successfully cloned into *E. coli* expression vector. The crude recombinant T1 lipase had an optimal working temperature and pH of 65 °C and 9, respectively. The lipase is also active over a wide pH range (6–11) and stable at 70 °C for up to 24 h.[18,19] The robust nature of the T1 lipase suggests that it is a good candidate for use in commercial ester syntheses that entail harsh processing conditions. We would like to highlight that it is our first attempt to utilize the thermostable T1 lipase as an alternative and feasible option for a solvent-free synthesis of menthyl butyrate. We hoped the crude T1 lipase was sufficiently efficient as the purified lipase in catalyzing synthesis of ester as it considerably reduces the processing steps of the lipase prior to use and hence, is less time consuming. The objectives of this study were to investigate the effects of the incubation time, temperature, enzyme loading and substrate to molar ratio, as well as to utilize RSM to determine the optimum conditions for the T1 lipase-catalyzed reactions and achieve maximum yield of menthyl butyrate.

## Materials and methods

### Materials

All chemicals were purchased from Sigma-Aldrich, USA. The recombinant T1 lipase was revived from stock culture in our lab and grown in Luria Bertani (LB) broth supplemented with antibiotics ampicillin and chloramphenicol.[18,20] The cultures were centrifuged at 10,000 rpm for 10 min and resuspended in distilled water before sonication. The crude lipase solution was centrifuged again under the same conditions and was adjusted to pH 9.0 using NaOH 2M prior to freeze drying and was kept at −20 °C.

### T1 lipase-catalyzed esterification

Menthyl butyrate was produced by esterification of menthol with butyric anhydride using lyophilized crude T1 lipase as the biocatalyst. The esterification reaction was carried out in screw-capped flasks (25 mL) and each set of reaction was prepared in triplicates. The lyophilized crude lipase was weighed and added to menthol. The enzymatic esterification was started by addition of varying amounts of butyric anhydride with different molar ratio (mmol menthol/mmol butyric anhydride) to the reaction mixture. The reaction mixture was stirred at 200 rpm at the desired reaction temperature under different period of times. The coded and actual levels for the central composite rotatable design (CCRD) for variables generated by RSM are indicated in Table 1. For removal of generated water, 10 mg of 0.3 nm molecular sieves, previously dried overnight at 60 °C, were added 1 h after incubation started. A control without enzyme was run in parallel under the same conditions.

**Table 1.** Coded and actual levels of variables for the central composite rotatable design.

Variable	Levels				
	−2	−1	0	+1	+2
*A*: temperature (°C)	30.0	37.5	45.0	52.5	60.0
*B*: reaction time (h)	10	15	20	25	30
*C*: enzyme amount (mg)	2.0	2.5	3.0	3.5	4.0
*D*: substrate molar ratio (MR)	1	1.5	2.0	2.5	3.0

### Analysis and characterization of menthyl butyrate

The reaction was terminated by dilution with 5 mL isooctane and the solution was centrifuged at 10,000 rpm for 5 min to remove the enzyme. A sample of 1 mL of the solution was sent for gas chromatography analysis on a Perkin Elmer (model Clarus 600, USA) instrument equipped with flame-ionization detector and Ultra 1 capillary column (25 m × 0.25 mm i.d. × 25 μm film thickness). The temperature program was chosen as follows: 110–150 °C (4 °C/min) and 200 °C (5 °C/min). The injector and detector temperatures were 230 and 250 °C, respectively. The carrier gas, N_2_, flow rate was 1 mL/min and the split was 50:1. The composition of the reaction mixtures was calculated from the number of millimoles of produced menthyl butyrate.

### Experimental design, statistical analysis and optimization

A four factor five-level CCRD that required 30 experiments was used in this study. The variables and their levels selected for the synthesis of menthyl butyrate were as follows; *A*: temperature (40−60 °C), *B*: incubation time (10−30 h), *C*: amount of enzyme (2−4 mg) and *D*: molar ratio of menthol/butyric anhydride (1:1−3:1). The experiments were randomized for statistical reasons and each experiment was run in triplicates.

A software package, Design Expert Version 6.0.6 (Stat-Ease, Statistics Made Easy, Minneapolis, MN, USA) was used to fit the second-order model to the independent variables using the following Equation (1):(1) 
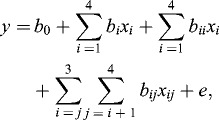
where *y* is the dependent variable (% yield) to be modelled; *x_i_* and *x_j_* are the independent variables (factors), *b*_0_, *b_i_* , *b_ii_* and *b_ij_* are the regression coefficients of the model and *e* is the error of the model. Analysis of variance (ANOVA) was used to determine the adequacy of the constructed model to describe the observed data. *R* ^2^ presents statistical points to the percentage of the variability of the optimization parameters explained by the model. Three-dimensional surface plots were generated to illustrate the main and interactive effects of the independent variables on the dependent ones.

## Results and discussion

### Model fitting and analysis of variance (ANOVA)

RSM consists of an empirical modelization technique used to evaluate the relation between experimental and observed results.[21] In order to obtain a proper model for optimization of menthyl butyrate synthesis in a solvent-free system, the CCRD was chosen as it has been widely accepted as the best design for response surface optimization.[22] The selected model consists of four factors and five levels including temperature, reaction time, amount of enzyme and substrate molar ratio. The experimental and predicted results are indicated in the matrix design (Table 2). The predicted values were acquired through model-fitting technique using the Design Expert software. Fitting of the data to various models (linear, two factorial, quadratic and cubic) and their subsequent ANOVA showed that the reaction was most suitably described with a quadratic polynomial model. The quadratic polynomial model, expressed in coded variables, is represented by the following Equation (2):(2) 
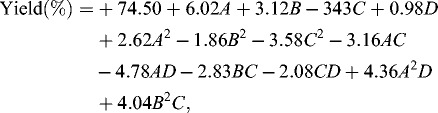

**Table 2.** Composition of the various runs of the central composite rotatable design, actual and predicted responses for the crude T1 lipase.

	Variable					
Run no.	Temperature (°C)	Time (h)	Enzyme amount (mg)	Molar ratio	Actual yield (%)	Predicted yield (%)
1	37.50	15.00	2.50	1.50	37.96	43.74
2	52.50	15.00	2.50	1.50	76.40	71.67
3	37.50	25.00	2.50	1.50	60.48	55.64
4	52.50	25.00	2.50	1.50	83.06	83.57
5	37.50	15.00	3.50	1.50	63.34	61.10
6	52.50	15.00	3.50	1.50	73.16	76.39
7	37.50	25.00	3.50	1.50	64.19	61.69
8	52.50	25.00	3.50	1.50	78.75	76.98
9	37.50	15.00	2.50	2.50	67.70	68.15
10	52.50	15.00	2.50	2.50	78.01	76.95
11	37.50	25.00	2.50	2.50	85.25	80.05
12	52.50	25.00	2.50	2.50	86.32	88.85
13	37.50	15.00	3.50	2.50	75.99	77.19
14	52.50	15.00	3.50	2.50	75.10	73.35
15	37.50	25.00	3.50	2.50	78.04	77.78
16	52.50	25.00	3.50	2.50	76.40	73.94
17	30.00	20.00	3.00	2.00	70.78	72.95
18	60.00	20.00	3.00	2.00	95.93	97.04
19	45.00	10.00	3.00	2.00	62.90	60.83
20	45.00	30.00	3.00	2.00	67.96	73.32
21	45.00	20.00	2.00	2.00	65.41	67.05
22	45.00	20.00	4.00	2.00	51.68	53.32
23	45.00	20.00	3.00	1.00	72.75	72.55
24	45.00	20.00	3.00	3.00	76.66	76.46
25	45.00	20.00	3.00	2.00	69.90	74.50
26	45.00	20.00	3.00	2.00	75.58	74.50
27	45.00	20.00	3.00	2.00	73.72	74.50
28	45.00	20.00	3.00	2.00	72.18	74.50
29	45.00	20.00	3.00	2.00	73.42	74.50
30	45.00	20.00	3.00	2.00	78.51	74.50

where *A* is the reaction temperature, *B* the time, *C* is the amount of enzyme and *D* the substrate molar ratio. In ANOVA, the *F-*value is derived from the ratio of regression mean sum of squares and error mean sum of squares (the difference between the predicted and experimental values), while the *P-*value refers to the probability value for the corresponding *F-*value. For the model fitted, the software generated model coefficients, *F-*values and *P-*values (Prob. > *F*) which points to the insignificant probabilities and was used to justify the significance of each experimental variable. If the *P-*value is very small (less than 0.05), this indicates that the individual terms in the model have a significant effect on the response. The computed *F-*value (15.96) for the model (Table 3) is significantly higher as opposed to tabulated *F*_13,16_ = 2.40 at *P* = 0.05, as well as a very small *P-*value (<0.0001) which implies the model is highly significant. This is further corroborated by a suitable coefficient of determination (*R^2^* = 0.9284) which shows more than 92% of variability for the conversion (%) can be explained by the model.

**Table 3.** Analysis of variance (ANOVA) and model coefficients obtained from the synthesis of menthyl butyrate catalyzed by crude T1 lipase.

Source	Sum of squares	Degree of freedom	Mean square	*F*-value	*P*-value
Model	3107.52	13	239.04	15.96	< 0.0001
*A* – temperature	870.61	1	870.61	58.11	< 0.0001
*B* – time	234.06	1	234.06	15.62	0.0011
*C* – amount of enzyme	94.26	1	94.26	6.29	0.0233
*D* – substrate molar ratio	7.64	1	7.64	0.51	0.4853
*A*^2^	192.79	1	192. 79	12.87	0.0025
*B*^2^	96.58	1	96.58	6.45	0.0219
*C*^2^	358.55	1	358.55	23.93	0.0002
*AC*	159.71	1	59.71	10.66	0.0049
*AD*	366.24	1	366.24	24.45	0.0001
*BC*	127.97	1	127.97	8.54	0.0100
*CD*	69.26	1	69.26	4.62	0.0472
*A*^2^	101.59	1	101.59	6.78	0.0192
*B*^2^*C*	87.24	1	87.24	5.82	0.0282
Residual	239.70	16	14.98		
Lack of fit	196.41	11	17.86	2.06	0.2192
Pure error	43.29	5	8.66		
Cor total	3347.22	29			

**Figure 1.**
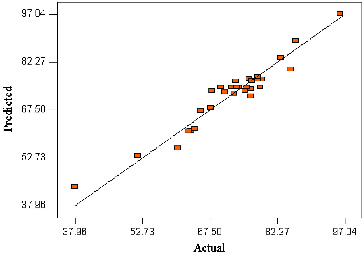
Comparison between the predicted and experimental values for the conversion of menthyl butyrate catalyzed by crude T1 lipase.

According to the ANOVA of factors, the *F-*value for the lack of fit is 2.06 is lower than the tabulated value of *F*_0.05(11,5)_ = 4.07, implying the lack of fit is not significant relative to the pure error, therefore, the relationships of the reaction parameters represented by the model are well within the ranges selected.[23] To evaluate the optimization technique, the observed and predicted values of the percentage conversion was compared and the results are depicted in Figure 1. As can be seen, the predicted values of the response from the model agreed well with the experimental values. Consequently, this model could be appropriately applied to navigate the design space.[24] Chaibakhsh et al. also reported a response model that sufficiently depicted reaction parameters in the enzymatic solvent-free synthesis of the commercial adipate ester.[8]

### Mutual effects of factors on the conversion of menthol

**Figure 2.**
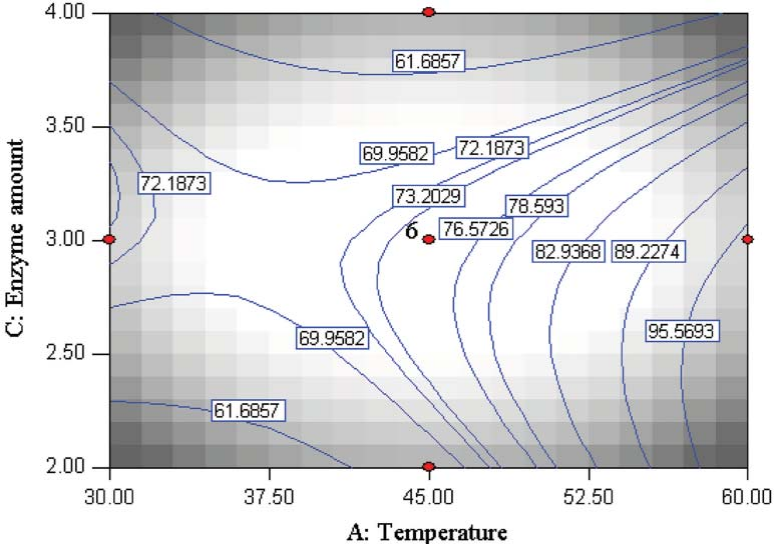
Contour plot showing the effect of temperature (*A*) and enzyme amount (*C*) and their mutual interaction in the synthesis of menthyl butyrate catalyzed by crude T1 lipase at constant molar ratio butyric anhydride/menthol (2:1) and time (20 h).

The contour plots illustrated the main and interactive effects of the independent variables on the conversion of menthol. Figure 2 depicts the effects of temperature and enzyme amount, as well as their mutual interaction in the synthesis of menthyl butyrate under constant conditions, substrate molar ratio (2:1) and time (20 h). As indicated by the linear coefficients and *F*-value of the parameters, temperature has a very significant influence on the percentage of conversion, and the response expected was a monotonic increase in conversion with increase in temperature. In other words, a high reaction temperature was more favourable in improving the conversion percentage. According to the *F-*value (Table 3), the effect of temperature on conversion was more significant than the amount of enzyme. It was observed that the percentage conversion increased with the temperature increase and peaked at 51.6 °C. Increasing the reaction temperature obviously improved substrate conversion due to greater unfolding of the enzyme [23] and the molecules become less rigid.[19,25,26]

It is widely accepted that temperature has two important roles in any reaction system. It has been described that an increase in temperature can reduce mixture viscosity that enhances integration of reactants. The diffusion process is also improved as mass transfer limitations are reduced, thereby favouring interactions between enzyme molecules and reactants.[27] Furthermore, crude T1 lipase has a working temperature of 65 °C [1] but has been known to catalyze reactions at 70 °C for prolonged periods of incubation with little enzyme inactivation. The higher temperature accelerates enzyme–substrate collision, consequently enhancing the conversion of substrates.[28] The crude T1 lipase achieved the highest conversion when 2.90 mg (0.60%, w/w) of enzyme was used. It was noted that any increase in enzyme loading beyond this point led to the decline of the product. This could be due to presence of superfluous enzyme molecules in the reaction mixture without involvement, thereby limiting diffusion and mass transfer.[12,28]

**Figure 3.**
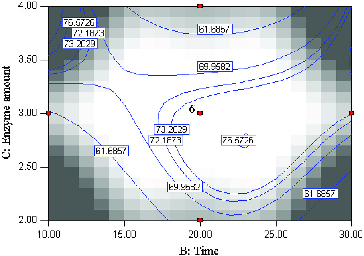
Contour plot showing the effect of time (*B*) and enzyme amount (*C*) and their mutual interaction in the synthesis of menthyl butyrate catalyzed by crude T1 lipase at constant molar ratio butyric anhydride/menthol (2:1) and temperature (45 °C).

Figure 3 shows the effect of variating reaction time and enzyme amount on the synthesis of menthyl butyrate at constant temperature 45 °C and substrate molar ratio (2:1). Prior to optimization, the range of reaction time was carefully chosen, otherwise, the optimal condition of synthesis could not be ascertain within the experimental region through the analysis of statistics and contour plots.[24] According to the *F-*value, the effect of time for the conversion of menthol is more significant than the amount of enzyme used. Reaction with fairly low enzyme amount (2.6 mg) and longer reaction time (22.5 h) resulted in 76.6% conversion of menthol. This observation is referred to as the inverse proportionality between reaction time and enzyme amount, as previously reported in many industrial enzyme-catalyzed processes.[8] Generally, minimal use of enzyme for high conversion of product is desirable as cost of enzymes can be quite high. In this study, reactions with low enzyme amount and short reaction time gave the lowest conversion of menthol.

**Figure 4.**
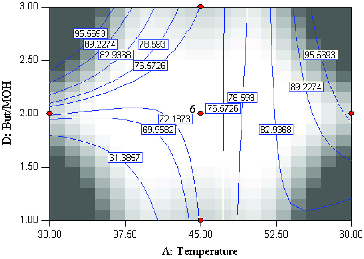
Contour plot showing the effect of temperature (*A*) and substrate molar ratio (*D*) and their mutual interaction in the synthesis of menthyl butyrate catalyzed by crude T1 lipase at constant enzyme amount (3 mg) and time (45 h).

The mutual interactions of varying temperature and substrate molar ratio under constant conditions, enzyme amount (3 mg) and time (20 h) are shown in Figure 4. According to the *F-*value, the effect of substrate (butyric anhydride:menthol) molar ratio is less important as compared to enzyme amount, reaction time and temperature. However, the interaction of temperature and substrate molar is a significant term due to a very small *P-*value (0.0001). The effect of temperature is very significant (*F-*value = 58.11) as opposed to substrate molar ratio (*F-*value = 0.51). Increasing the temperature has a significant positive effect on percentage conversion of the product. A high conversion of menthyl butyrate corresponding to 97.3% at 59.8 °C at substrate molar ratio 2.55:1 was observed. Interestingly, the contour plot also showed high percentage conversion at lower temperature coupled with high substrate molar ratio. This perhaps is due to the presence of larger amounts of substrate which increases the probability of substrate–enzyme collision.[12] As expected, the lowest product conversion occurred at low reaction temperature and low substrate molar ratio.

**Figure 5.**
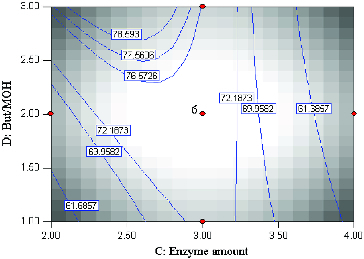
Contour plot showing enzyme amount (*C*) and substrate molar ratio (*D*) and their mutual interaction in the conversion (%) of menthyl butyrate catalyzed by crude T1 lipase at constant time (20 h) and temperature (45 °C).

From the economic point of view, it is desirable to achieve high percentage conversion of substrate at low enzyme amounts and high substrate levels as this offsets the usually high cost of enzymes. The following illustrates the effect of enzyme amount and substrate molar ratio under constant reactions conditions, temperature 45 °C and time 20 h (Figure 5). According to the *F-*value, the effect of enzyme amount was more significant than substrate molar ratio. A high percentage conversion was achieved with high substrate levels and low enzyme amounts. The highest conversion was accomplished with enzyme amount of 2.43 mg (0.39%, w/w) and substrate molar ratio (2.99:1).

### Attaining optimum conditions and verification of the model

The highest yield accomplished from the various runs was 95.93% using temperature 60 °C, 3 mg of enzyme, butyric anhydride/menthol molar ratio of 2:1 and reaction time 20 h. For development of industrial processes for the esterification of menthol for use in cosmetic formulations, food additives and medicines, the first aspect that comes to mind is reducing production cost. With regards to this, a high degree of conversion was possible by simply seeking the optimum point on the response surface. The software Design Expert 6.0.6 proposed several experimental conditions to find the optimum point and maximize percentage conversion under a variety of preferred conditions. However, only four sets of the predicted conditions suggested by the model were chosen. The first two were chosen for the highest percentage conversion of product under conditions of minimum enzyme amount and shortest reaction time. The last two experimental sets were options for the shortest reaction time, whereby the enzyme amount was not specified but within the range of the model. The experiments using the proposed conditions were performed and the observed results are shown in Table 4. The experimental values were found to be reasonably close to the values predicted by the model, therefore, confirming the adequacy and validity of the predicted model. T1 lipase was able to catalyze esterification of menthol to menthyl butyrate with percentage conversion close to 100% employing fairly low enzyme amounts and low reaction time. Thus, it has been demonstrated that RSM can be applied effectively to predict conditions for high conversion of menthyl butyrate catalyzed by T1 lipase. The enzyme catalyzed high conversion of menthyl butyrate close to completion under optimized operating conditions generated by CCRD.

**Table 4.** Optimum conditions for the crude T1 lipase-catalyzed synthesis of menthyl butyrate.

Experiment	Temperature (°C)	Time (h)	Enzyme amount (mg)	Substrate molar ratio (But/MOH)	Predicted yield (%)	Actual yield (%)	Deviation (%)
1*	60.00	11.37	2.55	3.00	98.30	97.4	0.9
2*	60.00	13.15	2.53	2.70	99.17	99.3	1.9
3	59.76	10.00	3.03	2.99	100.00	98.5	1.5
4	59.94	10.02	3.00	2.96	99.99	97.5	2.5

Note: (*) denotes the optimum conditions for the highest proposed by the model for minimum time and enzyme amount. The ones without the asterisks were the conditions proposed for minimum time only to obtain high yield of menthyl butyrate.

## Conclusion

In this work, we demonstrated that the thermostable crude T1 lipase was an exceptionally robust and efficient biocatalyst to be used for synthesis of menthyl butyrate in a solvent-free system. The study found that variables such as reaction temperature and incubation time were the major factors that affected the yield of menthyl butyrate followed by enzyme amount and lastly, substrate molar ratio. The response contours revealed optimal combination of parameters to afford the highest conversion of the product at low incubation time and enzyme amount in a solvent-free system were 60 °C, incubation time of 13.15 h using enzyme load of 2.53 mg (0.53% w/w, enzyme/substrate) and substrate molar ratio butyric anhydride/menthol of 2.7:1. The predicted yield was 99.17%, closely agreed with the actual experimental value of 99.3%. While changing other conditions, with the use of short incubation time, it also resulted in a similar percentage yield of 98.5% and is in good agreement with the predicted of 100% conversion. Hence, our first attempt of using RSM for high yield synthesis of menthyl butyrate was successful using crude recombinant T1 lipase. It can be said that the T1 lipase is a promising option to overcome drawbacks related with solvent-assisted enzymatic reactions.
